# Intriguing Relationships Between Cancer and Systemic Sclerosis: Role of the Immune System and Other Contributors

**DOI:** 10.3389/fimmu.2018.03112

**Published:** 2019-01-10

**Authors:** Alexandre Thibault Jacques Maria, Léo Partouche, Radjiv Goulabchand, Sophie Rivière, Pauline Rozier, Céline Bourgier, Alain Le Quellec, Jacques Morel, Danièle Noël, Philippe Guilpain

**Affiliations:** ^1^Medical School, Montpellier University, Montpellier, France; ^2^Department of Internal Medicine-Multiorganic Diseases, Local Referral Center for Auto-immune Diseases, Saint-Eloi Hospital, Montpellier University, Montpellier, France; ^3^IRMB, INSERM, CHU Montpellier, Montpellier University, Montpellier, France; ^4^Department of Radiation Oncology, INSERM U1194/IRCM, ICM-Val d'Aurelle, Montpellier, France; ^5^Department of Rheumatology, Lapeyronie Hospital, Montpellier, France

**Keywords:** Systemic sclerosis (scleroderma), cancer, immunosurveillance, paraneopastic syndrome, immunoediting of cancer, autoimmunity

## Abstract

Systemic sclerosis (SSc) is an autoimmune connective tissue disorder, characterized by multisystem involvement, vasculopathy, and fibrosis. An increased risk of malignancy is observed in SSc (including breast and lung cancers), and in a subgroup of patients with specific autoantibodies (i.e., anti-RNA polymerase III and related autoantibodies), SSc could be a paraneoplastic syndrome and might be directly related to an immune response against cancer. Herein, we reviewed the literature, focusing on the most recent articles, and shed light onto the potential relationship between cancer and scleroderma regarding temporal and immunological dimensions.

## Introduction

Systemic sclerosis (SSc, or scleroderma) is a rare connective tissue disorder affecting middle-aged women and characterized by tissue fibrosis, vascular dysfunction (microangiopathy) and autoimmunity with the production of specific autoantibodies (1). Skin fibrosis (or scleroderma) is the hallmark of SSc and a leading cause of disability. According to the extent of skin thickening, two main phenotypes are described: limited or diffuse SSc. In diffuse SSc, multi-organ involvement (i.e., pulmonary fibrosis) is associated with poorer prognosis and shorter life expectancy. Unfortunately, there remains an unmet medical need in SSc, a peculiar autoimmune condition where usual immunosuppressant drugs lack efficacy (2).

With population aging, cancer incidence is continuously increasing and represents a major public health problem. In France for instance, in 2017, 400,000 new cases were estimated, including 214,000 men and 186,000 women (3). The most common cancers were prostate cancer followed by lung cancer and colorectal cancer in men. In women, breast cancer, followed by colorectal cancer and lung cancer were the most common cancers, with an increase in lung cancer incidence that tends to become the second cancer in frequency. On the whole, cancer caused about 150,000 deaths that year.

Many studies have shown an increased risk for malignancy in SSc Similar to the general population, an increase in the incidence of malignant tumors has also been noted in the last decades. Together with a better management of scleroderma-related complications due to medical progress in SSc, cancer has thus become a leading cause of mortality in this disease, resulting in about 11% of deaths (third cause) according to the study on death certificates EUSTAR database (4). As a consequence, overall mortality has not improved in SSc in the last four decades, and the management of cancer including early diagnosis and adapted treatment becomes a challenging issue in this disease (2, 5). If no consensual recommendation has been made to date, some authors have suggested screening strategies, based on the most recent data on identified risk factors in this disease (6, 7).

The association of SSc and cancer is not fortuitous and the temporal clustering observed in some patients raises the possibility of SSc as a paraneoplastic syndrome in some patients, as described in other autoimmune conditions such as dermatomyositis (6, 8). However, the connections between the two are more complex than once imagined, and may result from many and diverse mechanisms (9). While immunosuppressants used to treat this autoimmune condition may lead to cancer development (10, 11), cytotoxic anti-cancer therapies have been associated with the development of scleroderma-like features such as Raynaud phenomenon, digital ischemia and fibrosis (12–15). Moreover, common mechanisms and pathways may be involved both in fibrogenesis and oncogenesis, and recent data have suggested that autoimmunity in SSc may be triggered by antigen mutation in tumor cells (6, 16, 17). In that sense, the immune response observed in these patients could be considered as protective, enhancing anti-tumor defenses. More recently, the advent of immune checkpoint inhibitors to treat numerous cancer types, sometimes triggering autoimmune responses, underscores the importance of immunity in cancer emergence and spread. Altogether, a growing body of data points out the intriguing bilateral relationships between SSc and cancer. Herein, we reviewed the most recent literature on this subject, and shed light onto their relationships through epidemiological, biological, and immunological dimensions.

## Cancer in Systemic Sclerosis: Epidemiology

### General Aspects

Published studies demonstrated an increased risk of all cancers in SSc, but the associated factors and the increased risk according to cancer subtypes widely differs. This heterogeneity is probably related to the number of patients included, to different follow-up durations, and to the different methods of analysis used in each study. In the study by Wooten and coll. published in 2008, 3.6 to 10.7% of SSc patients had developed at least one cancer. Lung cancer was the most common, followed by breast cancer. The risk factors included female gender, older age, and cutaneous diffuse form of SSc (18).

In a Danish cohort of 2,205 SSc patients, 222 had cancer after the onset of SSc, a higher incidence than in the general population with a standardized incidence ratio (SIR) of 1.5 (95% CI 1.3–1.7) (19). In this cohort, male patients had a higher risk than female and lung cancer and lymphoma exhibited the highest incidence rates. Of note, there was no association between SSc and breast cancer.

Two meta-analyzes performed in 2013 provided additional information. The first one by Bonifazi et al. included 16 studies involving more than 7,000 patients (20). The relative risk of cancer in SSc patients was 1.75 (95% CI 1.41–2.18). There was a strong association with lung cancer with a relative risk (RR) of 4.35 (95% CI 2.08–9.09), and hematological disorders with a RR of 2.24 (95% CI 1.53–3.29). Again, the association with breast cancer was not confirmed.

The second meta-analysis by Onishi et al. involved more than 6,000 patients through six cohort studies and observed a SIR of 1.41 (95% CI 1.18–1.69) and a higher SIR in male than in female patients (1.85 vs. 1.33) (21). An increased SIR was observed for the following cancers: lung cancer (SIR = 3.18, 95% CI 2.09–4.85), malignant hematological disorder (2.57, 1.79–3.68), hepatocellular carcinoma (SIR = 4.36, 2.00–9.51), and bladder cancer (SIR = 2.00, 1.06–3.77). There was no significant difference between cutaneous limited and diffuse SSc.

Recently, Igusa et al. analyzed the cancer risk in subgroups according to SSc phenotype, autoantibodies, and temporal clustering (22). In this study involving 2,383 SSc patients, 205 patients also had cancer. Surprisingly, the overall risk of cancer was not found increased compared to the general population. However, an increased risk for malignancy was found in anti-RNA-Pol-III-positive patients (SIR = 2.84, 1.89–4.10) and sero-negative patients (SIR = 1.83, 1.10–2.86). In contrast, anti-centromere-positive patients had a lower risk of cancer (SIR = 0.59, 0.44–0.76). Interestingly, among anti-RNA-Pol-III-positive patients, diffuse SSc phenotype was associated with breast cancer, while limited SSc phenotype was associated with lung cancer. These data would allow stratification of cancer risk by clinical and serological phenotype and thus allow targeted screening in this population.

## Demonstrated Associations With Specific Cancers

### Lung Cancer

The association of lung cancer and SSc is widely documented in the literature with a SIR ranging from 4.2 to 5.9 (23–25). In a retrospective Italian study of 318 patients, 16 patients had lung cancer (5%) (26). There was an association with the male gender, a longer duration of the disease, a younger age at the diagnosis of SSc. Pulmonary fibrosis was a risk factor for lung cancer with an OR of 6.7 (95% CI 2.2.−20.7). Anti-Scl70 antibodies were also a risk factor (95% CI 1.7–24.1). Over the sixteen SSc patients with lung cancer, thirteen died because of lung cancer. Another study did not identify any increase in lung cancer incidence compared to the general population, but in this single-center study there was a significant number of lung cancer in their general population (27). Notably, both the existence and the duration of interstitial lung disease (pulmonary fibrosis) in SSc have been confirmed in numerous other studies as an independent risk factor for cancer (5, 28, 29). Various common mechanisms involving chronic inflammation, tissue remodeling, cell cycle, and the sequestration of carcinogens by fibrosis have been suggested as a possible explanation for this association (5, 6).

### Breast Cancer

The incidence of breast cancer in scleroderma patients is extremely variable and discordant according to the studies. This variability remains unexplained to date but is potentially related to study methods and inter-country heterogeneity (19, 30). In an Italian study on 318 scleroderma patients, a significant increase in breast cancer incidence compared with the general population was observed. The SIR was evaluated at 2.1 (95% CI 1.13–3.90) (26). An increased risk of breast cancer has been described mainly in other small cohorts of patients but one of the largest cancer studies (Danish cohort of 2,205 patients with systemic scleroderma, 222 with cancer) and two recent meta-analyses did not confirm these results (19–21). However, a close temporal relationship may be observed between SSc and breast cancer in a subgroup of patients. Indeed, there appears to be a short delay between breast cancer and SSc diagnosis, according to several case series or retrospective cohort studies. In a literature review, a majority of patients (61.4%) were diagnosed with cancer within a period from 1 year before to 1 year after SSc diagnosis (31). In the Italian study by Colaci, the median time between these two conditions was 2.5 years (26). These findings might suggest common pathogenesis pathways between these two diseases (6, 8, 15, 29).

Additionally, the group of SSc patients with breast cancer would exhibit some characteristics, such as the presence of lung fibrosis and the absence of anti-nuclear antibodies (32). Patients who developed breast cancer after the diagnosis of SSc had distinct characteristics from others: older age at the time of SSc diagnosis, more frequent interstitial pneumonia with lung fibrosis, and less frequent familial history of breast cancer. However, more recent studies have suggested a link between specific autoantibodies such as RNA Polymerase 3 (RNA-PolIII), PM/Scl or RNA binding Protein Containing 3 (RNPC) antibodies and breast cancer (7, 17, 33–35).

Concerning the association between SSc and breast cancer, two points should be underlined. First, the female susceptibility observed in SSc suggests influence of the same hormonal factors that are found involved in breast cancer. For instance, elevated prolactin levels and decreased levels of DHEA (dehydroepiandrosterone sulfate) were found in patients with SSc and in those breast cancer patients (36, 37). Secondly, calcium channel blockers (CCB), a cornerstone treatment for SSc vasculopathy, have been suspected as a risk factor for breast cancer in the general population in a case-control study (38). The long-term use of CCB (over 10 years) was associated with an OR of 2.3 (95% CI 1.2–4.9) for ductal carcinomas and an OR of 2.6 (95% CI 1.3–5.3) for lobular carcinomas. Another study showed that patients with CCB had an increased risk of aggressive breast tumors with an OR of 1.96 (95% CI 1.09–3.53) (39). Some authors therefore suggested that CCB are potentially a confounding factor that might explain the increased incidence of breast cancer in SSc observed in some studies (40). However, all these results have recently been questioned by an epidemiological study taking into account confounding factors and involving 28,000 patients (41). The possible pathophysiological relationship between breast cancer and CCB remains unclear, but a hypothetical mechanism might be an impaired functionality of intracellular calcium associated with CCB, particularly in the initiation of the pro-apoptotic signal.

### Esophageal Cancer

The standardized ratio of esophageal cancer incidence in scleroderma patients is estimated at 15.9 (95% CI 4.2–27.6) (42). This increased risk of esophageal cancer is undoubtedly related to the high frequency of chronic gastro-esophageal reflux disease (GERD), increasing the risk of Barrett's esophagus, and thus the risks of dysplasia and adenocarcinoma of the esophagus. Several studies focused on this risk, which was estimated at 3% per year for patients with Barrett's esophagus over a three-year prospective study from EULAR network centers (43, 44). Among 46 SSc patients who had Barrett's esophagus at baseline and completed the follow-up, four developed esophageal adenocarcinoma. Dysplasia at baseline was a major factor risk of cancer. These data prompt endoscopic follow-up of Barrett's esophagus in SSc.

#### Hematological Malignancies

The association between autoimmune diseases and lymphoma has been described in several studies (45). An epidemiological study in Denmark and Sweden of 25,000 patients with non-Hodgkin's lymphoma found an association with autoimmune diseases (including SSc) with an OR ranging from 1.6 to 5.4 (46). In another Swedish study, the risk of lymphoma in SSc patients was increased with an SIR of 2.1 (47). In these studies, associated Sjogren's syndrome was not always reported, although it remains an independent risk factor for the emergence of lymphoma.

#### Bladder Cancers

The use of high cumulative dose of cyclophosphamide may lead to the development of bladder cancer in SSc, as reported in various studies (5, 48).

### Suspected Associations With Other Cancers

Concerning gynecological malignancies, a Canadian study found a higher prevalence of cervical dysplasia in the scleroderma population than in the general population (25.4 vs. 13.8%), particularly in patients with diffuse SSc (49). In another study, overall frequency of human papilloma virus (HPV) was not higher in SSc patients than in general population (50). However, the high risk HPV52 was the most frequent genotype and a greater multi-HPV infection rate was observed in SSc, particularly in diffuse SSc. Interestingly, cyclophosphamide was reported as a risk factor for cervical intraepithelial neoplasia with a dose-dependent cumulative risk (plus 13% increased risk of cervical dysplasia per each increase of one gram according to a series of patients with systemic lupus erythematosus) (51). These data prompt to screen carefully patients for whom immunosuppressive therapy is indicated.

Autoimmune thyroiditis is a condition commonly associated with SSc. The percentage of patients with hypothyroidism varies from 2.4 to 26% and the percentage of anti-TPO antibodies varies from 12 to 52% according to the studies (52). Some cases of scleroderma patients with thyroid cancer have been reported. In a study of 769 patients with SSc, no significant association was found and only 2 cases of thyroid cancer were observed (42). Another recent study suggested an increase in thyroid papillary carcinomas in SSc patients but all those patients with cancer had autoimmune thyroiditis (53). Once again, chronic inflammation might promote cancer development in this condition.

Rare cases of cutaneous squamous cell carcinoma have been reported in scleroderma patients, mostly associated with localized scleroderma (morphea, including pansclerotic morphea), (5, 54). Intriguingly, some patients with melanoma treated with interferon or immune checkpoint inhibitors have developed SSc (55–57).

Interestingly, a few cases of soft tissue malignant tumors (i.e., sarcomas) have been described in association with SSc, although no statistical link can be established, mainly because of the rarity of both conditions (58–64). This association between connective tissue disease and malignancy highlights the possibility of common mechanisms between the loss of connective tissue homeostasis in SSc and oncogenesis in sarcoma, including angiogenesis defects.

We summarized the demonstrated and putative associations between SSc and cancer in a table (see Table 1).

**Table 1:** Associations between systemic sclerosis and cancer: evidence from epidemiological data and suspected risk factors.

**Cancer**	**Evidence for association**	**Mean SIR**	**Scenario**	**Comments**
Lung	YES	4–6	A,B	There is a strong association between lung cancer and SSc in the presence of interstitial lung disease and anti-scl-70 Ab. Chronic inflammation in PF may lead to tumor development.
Breast	YES	2–3	B,C	Breast cancer is the most frequent cancer found in SSc patients. The risk of developing breast cancer in SSc is associated with the presence of anti-RNA-PolIII Ab through a pathophysiological process leading to paraneoplastic autoimmunity. CCB could also contribute to the development of breast cancer.
Esophageal	YES	15	A	GERD, a hallmark of SSc, is responsible for chronic inflammation in esophagus, and can lead to Barrett's esophagus, dysplasia, and adenocarcinoma.
Bladder	YES	2	C	The use of cyclophosphamide in SSc patients can lead to the development of hemorrhagic cystitis and bladder cancer through a cumulative dose-effect.
Hematological	YES	2	A	An increased risk of lymphoma has been described in numerous autoimmune diseases, including SSc, and may be more specifically associated with the presence of secondary SjS.
Cervix	NO	N/A	C	An increased frequency of high-risk HPV infection has been reported in SSc. The use of cyc may lead to the development of cervical neoplasia in these patients.
Thyroid	NO	N/A	A	Thyroiditis, an autoimmune condition frequently associated with SSc may lead to chronic inflammation of the thyroid and the development of papillary carcinoma.
Skin	NO	N/A	A	Cases of squamous cell carcinoma have been reported in association with morphea, (i.e., localized scleroderma), a condition where chronic tissue inflammation may lead to cancer development.
Sarcoma	NO	N/A	A,B	Few cases of soft tissue malignant tumors (i.e., sarcomas) have been described in association with SSc. No statistical link can be established because of the rarity of both conditions.

*Scenario A: SSc leads to cancer development (via chronic tissue inflammation); Scenario B: suspected paraneoplastic phenomenon between SSc and cancer onset; Scenario C: SSc treatments favor cancer development*.

*SIR, standardized incidence ratio; PF, pulmonary fibrosis; Ab, antibody; CCB, calcium channel blockers; GERD, gastro-esophageal reflux disease; cyc, cyclophosphamide; SjS, Sjogren's syndrome; HPV, human papilloma virus*.

### Chronic Inflammation, Tissue Homeostasis, Fibrosis and Cancer Development

SSc cannot be considered as a systemic inflammatory disease such as systemic vasculitides or inflammatory bowel diseases. However, some locations of the disease (in particular, esophagus and lungs) are exposed to chronic tissue inflammation, which is considered nowadays as a major factor leading to the development of cancer. At least 25% of cancers might be related to persistent inflammation or chronic infection (65). In fact, inflammatory mediators, such as pro-inflammatory cytokines and free radicals, could induce genetic and epigenetic modifications, leading to alterations in the cell signaling pathways and thus breaking normal cell homeostasis. For example, free radicals can randomly induce point mutations in tumor suppressor genes and contribute to the progression of cancer (66–68).

According to this concept, some authors proposed that chronic tissue damage with recurrent tissue repair mechanisms may be one of the mechanisms for the development of lung and esophageal cancer in SSc patients with pulmonary fibrosis and GERD, respectively (15). This phenomenon is well documented for SSc patients with chronic GERD, since these patients exhibit an increased risk of Barrett's esophagus, high-grade dysplasia and esophageal cancer. Similarly, chronic inflammation within fibrosis could contribute to cancer development in SSc patients with lung fibrosis (5, 15).

It is noteworthy that since SSc may be associated with other connective tissue disorders or organ-specific autoimmune diseases, patients may be exposed to an increased specific risk of cancer through these additional autoimmune conditions and their related-chronic inflammation. Notably, primary biliary cirrhosis (whose association with SSc represents “Reynolds syndrome”) and autoimmune thyroiditis (Hashimoto's) are associated with increased risk of cholangiocarcinoma (69) and papillary thyroid carcinoma respectively (53).

Another point to consider is fibrosis itself, as a condition potentially associated with cancer. Indeed, the association between pulmonary fibrosis and cancer is well known, especially in patients with idiopathic pulmonary fibrosis (IPF), which represents the key condition of lung fibrosis. About one in ten patients with IPF will develop lung cancer during follow-up (70). Apart from the role of chronic inflammation in pulmonary carcinogenesis, carcinogenic molecules may be sequestrated by fibrosis, secondary to an altered lymphatic drainage (71, 72).

Moreover, mesenchymal cells are crucial for the development of cancer (73). This is well documented in lung cancer, in which tumor-associated fibroblasts contribute to a “growing” loop with malignant epithelial cells, through the production of specific growth factors such as epidermal growth factor (EGF), fibroblastic growth factor (FGF), or transforming growth factor (TGF). In this context, epithelial–mesenchymal transition (EMT) contributes to the development and spread of tumor cells, and is also a source of fibroblasts. This complex process including numerous phenotypic transitions toward mesenchymal cells (i.e., fibroblasts) may thus be potentially implicated both in cancer and fibrosis. On the one hand, EMT has been clearly observed during the development of lung cancer, when epithelial cells transform into malignant cells under the activation of oncoproteins such as mutant Kras (Kirsten rat sarcoma viral oncogene) (74, 75). Thus, EMT may participate to epithelial cell plasticity and modify their properties in the context of lung injury. On the other hand, EMT also represents a potential mechanism in SSc, where epidermal cells may acquire mesenchymal and fibroblastic features under the activation of TGF-beta signaling pathway within lesional skin, further increasing the fibrotic burden (76).

## Shared Mechanisms in Cancer and Systemic Sclerosis

### Genetics and Epigenetics

Common features may be observed in cancer and scleroderma, with common actors promoting disease development. Strikingly, a recent gene profiling study revealed oncogenic gene patterns in SSc (77). As abovementioned, the implication of EMT in both diseases is particularly interesting and may originate from common genetic and epigenetic alterations, involving telomere shortening, chromosomal instability, senescence, increased proliferation rates, immune deregulation, and impaired cell metabolism.

#### Telomere Shortening

While telomere maintenance is complex as well as essential for cancer progression (78), deficient telomerase activity and telomere shortening have been both reported in SSc (79, 80). In another fibrotic condition, IPF, telomere shortening is also a potential contributor for the pathological process and reduction in telomere length of circulating leukocytes could even have an impact on overall mortality (81).

#### Epigenetic Alterations

Epigenetic mechanisms could contribute to the pathogenesis of SSc, as a consequence of the exposure to environmental factors such as silica (see below paragraph 2.4.1). This would result in cytokine network modulation toward the development of autoimmunity. Similarly, an important body of data argues for the essential contribution of epigenetics to the development of cancer. Three main mechanisms should be considered with striking similarities between both diseases: changes in DNA methylation, histone modifications, and microRNAs.

First, a reduced expression of several genes regulating the process of methylation (such as DNA methyltransferase-1, methyl-CpG-binding domain 3, and MBD4) could induce DNA hypomethylation in CD4+ T cells in SSc (82, 83). Recently, Lian et al. observed an overexpression of CD40L in CD4+ T cells from female SSc patients (but not from male patients), which could be explained by a demethylation of specific CpG–DNA within regulating regions of CD40L (84). This overexpression of CD40L may be of great importance in SSc, since the interaction CD40/CD40L play key roles in autoimmune diseases, in particular in SSc. The demethylation within CD40L gene could result in the reactivation of the inactive X chromosome, maybe contributing to the female susceptibility of the disease. Other alterations in the methylation patterns have been observed in SSc. Endothelial cells are concerned by such epigenetic changes, since CpG hypermethylation of the bone morphogenetic protein receptor factor type 2 (BMPR2) was recently reported. Hypermethylation resulted in downregulation of the protein, inducing an increased sensitivity of endothelial cells to apoptosis and oxidative stress (85). Another example is represented by the hypomethylation of TNFSF7, the promoter of CD70, observed in SSc, which results in the overexpression of CD70 by CD4+ T cells (86). In fact, CD70 is a B cell co-stimulatory molecule, whose overexpression may contribute to autoimmunity, as observed in other autoimmune diseases like lupus and Sjögren's syndrome. So, CD70 hypomethylation could contribute to autoimmune diseases whereas hypermethylation would be rather associated with the development of malignancies, in particular breast cancers (87). Last but not least, SSc fibroblasts also appear to be affected by methylation changes. Indeed, hypermethylation of the CpG rich regions in the Fli-1 (Friend leukemia virus-induced erythroleukemia-1) promoter region was demonstrated in fibroblasts and skin from SSc patients (88). Interestingly, the latter also plays an important role in cancer development and appears as a possible link between autoimmunity and malignancy (89). Furthermore, in an experimental animal model of bleomycin-induced lung fibrosis, the regulatory effects of the methyl CpG binding protein 2 (MeCP2) and its implication in tissue homeostasis have been shown. The authors demonstrated that MeCP2 binds to the methylated region of α-SMA gene promoter and activates its expression (90). They then studied the effects of a loss of expression of MeCP2, using siRNA or knockout mice. Resulting in the methylation of α-SMA promoter, they observed less activation of this gene. Hence, MeCP2 deficient mice were less sensitive to bleomycin, highlighting the pivotal role of MeCP2 in myofibroblast transformation and fibrosis development.

Second, impaired transcription of genes and deregulated gene expression induced by histone modifications may also contribute to the development of both cancer (91) and SSc (83). Notably, novel therapies targeting epigenetic contributors such as histone demethylase and histone deacetylase inhibitors have been developed in the field of oncology and may emerge in the field of pulmonary fibrosis (92). In SSc, a recent study highlights the role of the histone acetyltransferase p300 in the development of fibrosis (93). In this study, increased p300 levels within SSc skin under TGF-beta activation resulted in collagen transcription in a Smad-independent manner involving Egr-1 (early growth response 1). Actually, p300-mediated histone H4 hyperacetylation appears to be a pivotal epigenetic modification in SSc, whose deleterious profibrotic effects could be counteracted by the blocking effects of antiaging deacetylase enzyme sirtuin 1 (SIRT-1) on Smad-dependent transcription (94). SIRT-1 belongs to the group of histone deacetylases called sirtuins (SIRTs) that includes 7 proteins (from SIRT1 to SIRT7) implicated in the regulation of aging process, clock control, and cell metabolism. Interestingly, in this study, the expression of SIRT-1 was found reduced in SSc skin and its activation by resveratrol reversed the fibrotic response of fibroblasts (94). Emerging data corroborate this observation in SSc (95) and also suggests the role of SIRTs proteins in the development of cancer (96). Altogether, SIRTs proteins could contribute to SSc and cancer through different mechanisms: TGF-β signaling, mTOR pathway, oxidative stress and cellular senescence.

Third, numerous microRNAs, including miR-21, miR-29 family and let-7d, have been reported to play key roles in the pathogenesis of cancer and fibrosis, and could even represent potential therapeutic targets in both diseases (77). In SSc, their effects concern collagen gene expression in fibroblasts, collagen degradation, thus extracellular matrix remodeling, apoptosis, and epithelial-mesenchymal transition. Firstly, miR-21 may function as an amplifying factor, enhancing TGF-β signaling events in SSc fibrosis (97). Exosomal miR-21 might be used as a cancer biomarker, and its combination with other miRNAs within a specific panel may become a relevant diagnosis tool for cancer (98). Notably, it has been associated with breast cancer. Secondly, the miRNA-29 family, consisting of miR-29a, miR-29b, and miR-29c, could also be involved in cancer development and fibrosis. miR-29b functions either as a tumor suppressor or an oncogene under specific conditions and could mediate cancer chemo-sensitivity or resistance. The miR-29 family members also appear to be antifibrotic mediators, modulating collagen expression and degradation. Indeed, the expression of miR-29a is reduced both in fibroblasts and skin in SSc (99, 100), and its overexpression induced a reduction of fibroblast proliferation and collagen synthesis *in vitro* (101). A similar reduced expression of miR-29a has also been found both in broncho-alveolar cells from IPF and lung cancer patients, suggesting a common link between the two conditions (102). Altogether, miR-21 and miR-29 family members exhibit synergistic functions to modulate fibroblast fate both in healthy and fibrotic conditions. Thus, an imbalance between these two mediators might contribute to fibrosis. Other miRNAs strongly involved both in cancer and fibrosis are miR-16 (77) and let-7d. The latter is considered as a key regulator of cell proliferation and can act as a tumor suppressor (103). It is also involved in the regulation of EMT and prevention of lung fibrosis (104). Its expression is reduced in SSc skin (105). The role of the miRNAs in cancer and scleroderma also underscores the importance of exosomes in both diseases, since miRNAs are contained and conveyed by such vesicles (106). Interestingly, anti-PM/Scl autoantibodies found in SSc and associated with malignancy recognize a complex that is the human homolog of *saccharomyces cerevisiae* exosome (35, 107). This further strengthens a pathological link between autoimmune response in SSc and cancer.

### Common Signal Transduction Pathways

Signal transduction pathways may also be shared by cancer and fibrosis, as highlighted by the recent gene profiling study by Dolcino et al. revealing oncogenic signature in SSc patients, involving numerous well-known oncogenic proteins such as Ras, janus kinase (jak), Avian Myelocytomatosis Viral Oncogene Homolog (c-myc), B-cell lymphoma (bcl-2), Myeloid differentiation primary response 88 (myd88), poly(ADP-ribose) polymérase (PARP), and the phosphatidylinositol 3-kinase/Akt (PI3K/Akt) pathway (77). Interestingly, the transcription factor Fra (Fos-related antigen) is also involved both in breast cancer (108) and in scleroderma (109).

The PI3K/Akt pathway is implicated in lung fibrosis, and has been documented in experimental *in vitro* and *in vivo* models and may be a target for therapy (110). The antagonist of this pathway, Protein Phosphatase and Tensin homolog (PTEN), is also implicated in SSc: its expression is reduced in skin fibroblasts from patients with cutaneous diffuse SSc. Furthermore, the gene deletion of PTEN in adult mouse fibroblasts induced skin thickening, activation of PI3K/Akt pathway and increased expression of connective tissue growth factor (CTGF/CCN2) (111), the latter mediator representing an essential actor in this model (112). The Wnt/beta-catenin pathway, strongly implicated in cancer development, has also been reported playing a role in lung fibrosis (113). An aberrant activation of this pathway would result in accumulation of beta-catenin within pulmonary tissue, promoting EMT. In the field of cancer, the dysfunction in Wnt pathway could favor cancer emergence and spread, but also resistance to anti-tumor treatments (including immunotherapy such as immune checkpoint inhibitors). Aberrant Wnt pathway could in fact induce deficient immunosurveillance toward cancer, leading to immune-evasion (114). The activation of the Wnt pathway was also observed in lesional skin from SSc patients. It stimulated a Smad-dependent fibrotic process in mesenchymal cells. It repressed adipogenesis in subcutaneous pre-adipocytes, while inducing myofibroblast differentiation (115, 116).

Some authors hypothesize that dysfunction of regulation of the TGFβ / SMAD pathway by caveolin-1 (Cav-1) is involved in both the development of fibrosis and breast cancer (117). In SSc, there is a decrease in Cav-1 expression in the lung and skin of SSc patients. KO mice for Cav-1 develop pulmonary and cutaneous fibrosis. *In vitro*, when Cav-1 function is restored in cultured SSc fibroblasts, their phenotype was normalized and stimulation of the TGFβ pathway was stopped by the inhibition of SMAD3 phosphorylation. Thus Cav-1 would allow the inhibition of SMAD3 phosphorylation and regulate the fibrosis process (118). In breast cancer, Cav-1 gene is a tumor suppressor gene, and a dominant negative mutation is present in 16% of breast cancers. Using a transgenic mouse model of breast cancer, the authors have demonstrated that cav-/- mice have a significant increase in tumor volume and rapidly develop pulmonary metastases, unlike cav+/+ or cav+/- mice (119).

On the whole, the oncogenic pathways found activated in SSc might contribute to fibrosis development while predisposing to malignancy in this disease.

### Environmental Factors and Treatments as Inducers of Both Diseases

#### Environmental and Occupational Exposures in SSc and Cancer

Crystalline silica is a known carcinogen for bronchopulmonary cancers and also an environmental factor involved in the development of SSc (120). Many other environmental components have been suspected or confirmed as playing a role in the emergence of SSc, such as organic solvents, pollutants, welding fumes, pesticides, etc (121–124). Some of them are recognized as carcinogens.

#### Immunosuppressant-Induced Cancers

Several immunosuppressive drugs can be used in SSc, as recommended by EULAR, but may contribute to cancer (125). Among them, cyclophosphamide is a well-known agent, which is able to promote bladder cancer with a dose-dependent relation (10, 11). In a retrospective study with control group, there was 4 times more cancer in the SSc population treated with cyclophosphamide, with a consequent higher number of hematological malignancies (126). Recent studies have suggested that mycophenolate mofetil (MMF) is an alternative to cyclophosphamide for the treatment of interstitial lung lesions, including improved tolerance (127, 128). However, few case series have suggested a possible relationship between squamous cell carcinoma and mycophenolate mofetil in scleroderma, with cancer regression after MMF withdrawal (129).

#### Anti-cancer Therapy-Induced SSc

Several scleroderma-like syndromes have been described after anti-mitotic treatment. Docetaxel, a molecule used in the therapeutic arsenal of many cancers, including breast cancer, is known to have skin toxicity, with possible scleroderma-like lesions. More than a dozen cases of patients with authentic limited or diffuse SSc have also been reported secondary to docetaxel. The mechanisms leading to docetaxel cutaneous fibrosis are not fully understood. Some authors suggest that the deposition of an extracellular matrix glycoprotein (i.e., versican) after docetaxel or paclitaxel treatment may play a role in the pathogenesis of docetaxel-induced scleroderma (130). Recently, the first case of scleroderma secondary to docetaxel with organ involvement (PAH and renal scleroderma crisis) has been published (131). One of the explanations would be the possibility of endothelial cell damage induced by oxidative stress secondary to docetaxel (132).

Other chemotherapies have been associated with tissue fibrosis and/or scleroderma, such as bleomycin and gemcitabine (12, 14).

Ionizing radiation may be responsible for morphea (133) and may exacerbate pre-existing systemic scleroderma, according to some authors (13, 134, 135).

Strikingly, graft versus host disease (GVHD), a common complication of allogeneic hematopoietic stem cell transplantation (HSCT), somehow mimics SSc (136). Indeed, is skin fibrosis is a hallmark of chronic GVHD, resembling scleroderma. Beyond common clinical features, shared pathways are involved as observed in preclinical models for the two conditions (137–139).

More recently, anticancer immunotherapy using immune checkpoint inhibitors has been shown to trigger autoimmunity, and a few cases of scleroderma have been reported in the literature (55–57, 140). In this context, the complexity of the interplay between anti-cancer pre-existing autoimmunity, genuine paraneoplastic syndromes and the effects of immune system stimulation by biologics is striking, and strengthens the relationships between SSc and malignancy.

## Autoantibodies as Biomarkers Predicting Malignancy in Systemic Sclerosis?

### Anti-RNA Polymerase III

In a monocentric retrospective study in England, out of 2,177 patients with SSc, 7.1% had a history of cancer (33). The frequency of cancers was significantly increased in patients with anti-RNA-PolIII (14.2%) compared to patients with anti-Scl70 (6.3%) and anti-centromere (6.8%) autoantibodies (*p* < 0.0001 and *p* < 0.001). In patients diagnosed with cancer within 3 years of SSc onset, 55.3% had anti-RNA-PolIII. In addition, SSc patients with anti-RNA-PolIII had twice as much risk of developing cancer as anti-centromere patients (*p* < 0.0001).

These data were confirmed by a study of EULAR, where anti-RNA-PolIII antibodies were associated with synchronous malignancy (-6 months + 12 months) with an OR estimated at 7.38 (95% CI 1.61–33.8) (7). The association with breast cancers was even stronger, with an OR of 20.2 (95% CI 1.45–355). Epidemiology and the shared role of sex hormones predisposing to both conditions may explain this specific association. In multivariate analysis anti-RNA-PolIII was also associated with older age, acute renal crisis and diffuse skin involvement. These results led to specific cancer-screening recommendations in anti-RNA-PolIII positive patients, over a period of 2 to 5 years, including routine mammography (repeated every year) and non-invasive investigations, such as prostatic specific antigen (PSA) testing, blood stool test, and gynecological examination. Of note, in an Australian prospective study, patients with anti-RNA-PolIII positive and anti-RNA-PolIII negative antibodies had the same percentage of cancer (13%) but the diagnosis of cancer within 5 years after the diagnosis of scleroderma was more frequent in anti-RNA-PolIII positive patients (13 vs. 3.9%) (141). A direct pathophysiological link between RNA-PolIII antigen modification in tumor and autoimmunity is even suggested in this condition, as developed below (paragraph Anti-RNPC-3).

### Anti-RNPC-3

Recent studies have identified in “triple negative” scleroderma patients with cancer (patients without anti-centromere, anti-scl70 and anti-RNA-PolIII antibodies), a new autoantibody that targets RNPC-3. RNPC-3 is a protein member of the minor spliceosome, ribonucleoprotein complex that participates in the splicing of pre-messenger RNAs (17). Like the anti-RNA-PolIII positive patients, these patients have 4 times more risk of developing cancer within 2 years after the onset of scleroderma than the anti-centromere positive patients (95% CI 1.1–16.9) (142). The authors also noted an association between anti-RNPC-3 and severe interstitial lung disease, as well as more frequent muscle involvement (142). These anti-RNPC3 autoantibodies would possibly be indicative of cancer-induced autoimmunity in this subgroup of patients.

### Anti-PM/Scl

A recent Spanish retrospective study involving 432 patients including 53 cancers (12.2%), found no association between anti-RNA-PolIII antibodiesand cancer (35). In contrast, the anti-PM/Scl autoantibody was associated with cancer with an OR of 3.90 (95% CI 1.31–11.61), while aspirin treatment was protective with an OR of 0.33 (95% CI 0.12–0.90) (35). As above mentioned, the link between miRNAs regulation and PM/Scl, a complex homolog to yeast exosome is intriguing. However, these results have not been confirmed by a Dutch study (143).

### Anti-Scl70

Rosen's team has shown that patients with anti-scl70 antibodies were associated with short cancer-scleroderma interval (34). Advanced age was an independent risk factor for cancer in this study. An Italian team has demonstrated that anti-Scl70 antibodies were associated with lung cancer (26).

## Paraneoplastic Scleroderma: From Epidemiological Observation to Pathophysiological Demonstration

### Overview of the Concept of Paraneoplastic Autoimmune Disease

As said earlier, the observation of an elevated risk of cancer in patients with numerous systemic autoimmune conditions compared with the general population argues for close and bidirectional relationships between malignancy and autoimmunity. Even more striking is the close temporal clustering in some patients, which leads to consider autoimmune disease as a genuine paraneoplastic entity. The exact definition of a paraneoplastic syndrome may vary greatly among authors and remains debatable. First, one prerequisite for paraneoplastic phenomenon lies in this “temporal clustering,” viz. a short interval between the onset of cancer and the onset of autoimmune disease. Of note, cancer could precede SSc onset, and vice-versa. This “short” interval is however vague, but 3 to 5 years before and after SSc diagnosis could be acceptable since it corresponds to a “peak” of frequency in cancer diagnosis in previous studies (33). Second, there should be a parallel evolution between cancer and autoimmunity, with autoimmune flares accompanying cancer relapses. Conversely, cancer resection should lead to the remission of the associated autoimmune condition. However, such a theoretical conception of paraneoplastic phenomena may be far away from the reality observed concerning SSc, where no resolution of autoimmunity has been described after cancer healing.

Conceptually, autoimmunity may be associated with oncogenesis and anti-tumor defense. This is also consistent with the observation of cancer developing some time after autoimmunity, since autoimmune response in this context may be triggered in the very early stages of cancer development, even in premalignant disease. For instance, autoantibody production has been shown to appear years before cancer diagnosis in such context (144). Long ago considered as non-specific, the presence of some antibodies could have some clinical relevance for patients developing autoimmune disorders and cancer, and become novel biomarkers for both conditions, in terms of early diagnosis, but also prognosis and response to therapy. Interestingly, specific anti-tumor activity of antinuclear antibody (ANA) *via* antibody-dependent cell-mediated cytotoxicity (ADCC) has been described (145). For some authors, antibody clustering could even correspond to distinct underlying malignancies, beyond autoimmune disease classification (145). For instance, in dermatomyositis, anti-NXP2 and anti-TIF1γ–but not anti-MDA-5 antibodies- are strongly associated with cancer development (more than 80% of patients presenting with cancer and dermatomyositis) (146).

Interestingly, and consistent with what is usually reported with paraneoplastic syndromes, autoimmunity in the context of cancer is most often associated with a better outcome. For instance, the presence of a paraneoplastic syndrome is associated with smaller tumors and less metastatic disease. Moreover, tumor-infiltrating lymphocytes, the presence of ANA and vitiligo are clearly demonstrated as positive prognostic factors (8, 145). In addition, in the context of cancer immunotherapy using immune checkpoint inhibitors (i.e., anti-PD1, -PDL1 and CTLA-4 agents), the presence of tumor infiltrating lymphocytes before treatment and the observation of autoimmunity (i.e., immune related adverse events) under treatment are usually predictive of a better response to therapy (147).

All these observations support the hypothesis of a common primary event in oncogenesis that could make a self-antigen become immunogenic, and next trigger an autoimmune response against tumor cells. This specific autoimmune response would contribute to anti-tumor defense, but in some extent, in the case of a shared antigen, this response could spread to non-mutated antigens and be responsible for healthy tissue damage in relation with autoimmunity. In SSc, this concept has recently been thoroughly demonstrated in patients with anti-RNA-PolIII antibodies and contributes to the possibility of scleroderma being a paraneoplastic disease.

### Cancer-Induced Scleroderma: The Role of Mutated RNA-PolIII in Autoimmunity

Several recent studies conducted by the team of Rosen, Casciola-Rosen and Shah (Johns Hopkins University School of Medicine, Baltimore, USA) among others, have shown that the anti-RNA-PolIII antibodies were associated with cancer in SSc, with a temporal clustering between the two conditions suggestive of a paraneoplastic disease and a common underlying mechanism (7, 16, 33, 141). Patients with anti-RNA-PolIII antibodies are indeed 5.08 (95% CI 1.60–16.1) times more likely to develop cancer within 2 years of SSc onset (34). Furthermore, a specific nucleolar expression of RNA-PolIII was observed in the malignant cells from these patients, suggestive of a link between cancer-related auto-antigen and autoimmune response (16).

In order to decipher the potential common pathophysiological process underlying this association, Rosen et al. comparatively studied eight tumors from anti-RPC1 positive patients (RNA polymerase III subunit) and eight tumors from anti-Scl70 or anti-cm positive patients. Genetic alterations (somatic mutation or loss of heterozygosity) of the POLR3A gene coding for RNA polymerase III were identified in the tumors of six out of eight anti-RCP1 positive patients, while no mutation of this gene was found in the tumors of anti-cm or anti-Scl70 positive patients. Hence, the presence of this mutated auto-antigen in malignant cells could be the *primum movens* triggering autoimmunity in these patients, inducing cellular and humoral responses, with the production of anti-RNA-PolIII autoantibodies (148). Of particular interest, anti-RNA-PolIII antibodies in these patients were found to recognize both mutated and non-mutated (wild-type, wt) RNA-PolIII. This important finding, observed in other situations (149), is related to an “epitope-spreading” mechanism, that would be responsible for healthy tissue damage in SSc. In other terms, their demonstration argues for the role of a shared antigen (i.e., RNA polymerase III) that could undergo genetic alterations (for instance under DNA oxidative damage), and lead to the emergence of a mutated and immunogenic antigen in transformed cells. These alterations would next trigger a mutant-specific clonal immune response that could subsequently spread to wt antigens in healthy cells, contributing to autoimmune-mediated tissue damage in SSc (Figure 2). According to this hypothesis, the appearance of scleroderma in the context of malignancy would be the “price to pay” for eliminating the cancer (6). This could also explain why 80% of RNA-PolIII patients never develop cancer: the latter ones may have benefited from anti-tumor immunosurveillance with efficient eradication of malignant cells. However, as summarized by Schreiber et al., immunosurveillance in cancer is a dynamic process, better-called immunoediting, that evolves in three stages, from tumor elimination, equilibrium, and finally to escape under tumor high mutational rate with loss of expression of immunogenic antigens(150).

## Conclusion and perspectives

**Figure 1:**
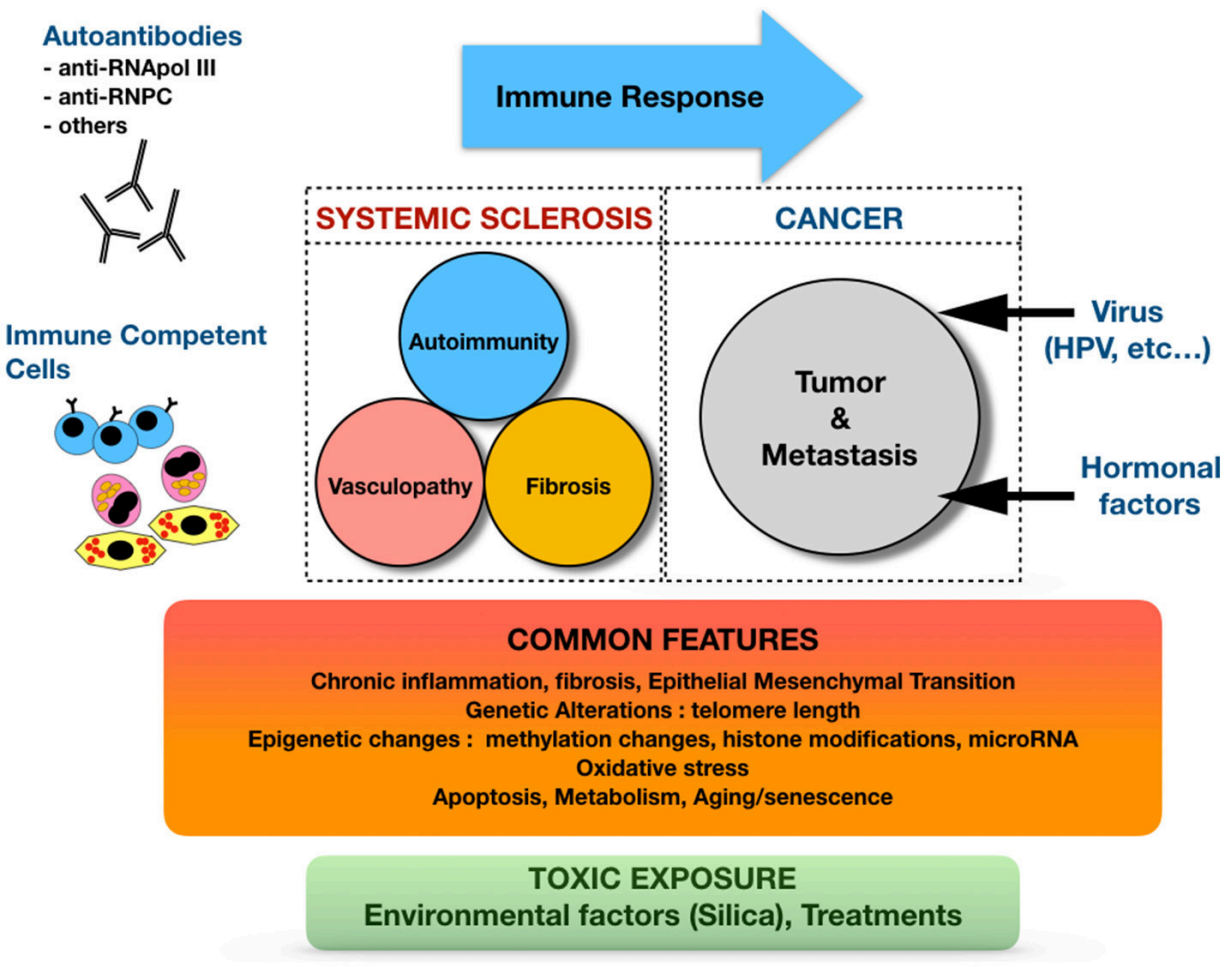
Complex and bilateral relationships between systemic sclerosis and cancer.

Beyond simple epidemiological observations, intriguing and complex bilateral relationships exist between SSc and malignancy, supported by a growing body of data involving the immune system and other contributors such as genetic and epigenetic changes, environmental factors, including oxidative stress. These relationships are summarized in Figure 1.

**Figure 2:**
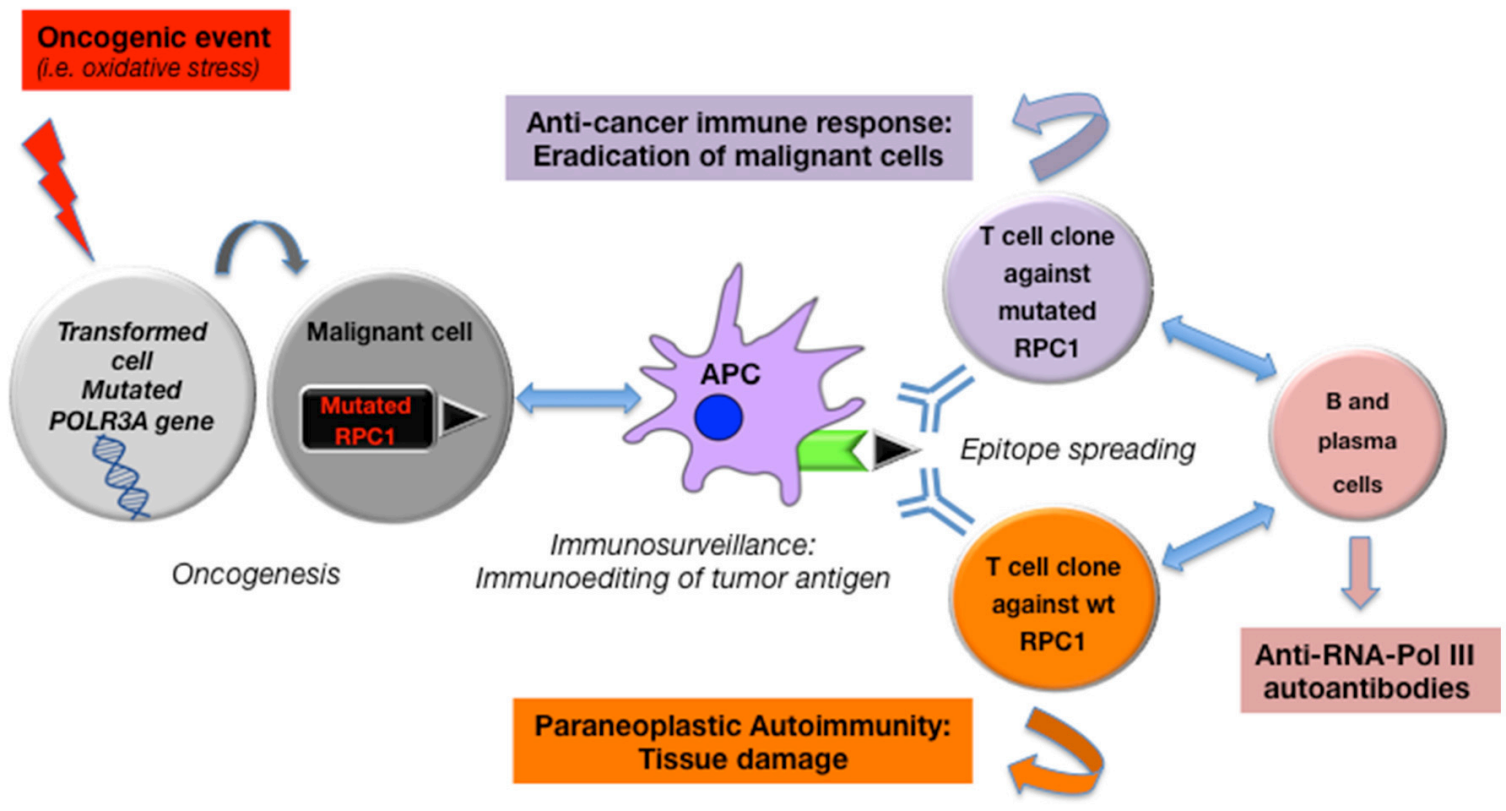
Anti-tumor driven auto-immunity: from mutated RNA polymerase III in cancer to paraneoplastic systemic sclerosis.

On the whole, based on these observations, a novel concept of autoimmunity as a response to underlying malignancy is coming up in scleroderma, and may contribute to new strategies in patients' care. Indeed, autoantibodies could be useful biomarkers for screening strategies, as proposed by some authors who postulate that early diagnosis of malignancy may ameliorate cancer but also SSc outcome (6, 7, 35). A detailed screening algorithm has been proposed by Shah et al., based on gender, specific risk factors and “red flags,” leading to specific non-invasive and invasive investigative procedures (6). Even if the beneficial effects in terms of overall survival in the scleroderma population remain to be demonstrated, a strategy based on repeated and more aggressive screening in patients with specific autoantibodies subsets (i.e., anti-RNA-PolIII, Pm/Scl or RNPC3) or in seronegative patients may be tantalizing.

Deciphering the mechanisms of autoimmunity through the prism of cancer immunosurveillance is even more fascinating in the era of anticancer immunotherapy, and will undoubtedly lead to new breakthroughs both in the field of autoimmunity and cancer. One could hope this may 1 day contribute to a better prognosis of scleroderma.

## Author Contributions

AM, LP, RG, and PG participated in the review of literature, and in the manuscript redaction. SR, PR, CB, AL, JM, and DN participated in the manuscript redaction and final approval.

### Conflict of Interest Statement

The authors declare that the research was conducted in the absence of any commercial or financial relationships that could be construed as a potential conflict of interest.
